# Physicians’ abilities to obtain and interpret focused cardiac ultrasound images from critically ill patients after a 2-day training course

**DOI:** 10.1186/s12872-020-01423-2

**Published:** 2020-03-30

**Authors:** Hongmin Zhang, Wei He, Hui Lian, Xiukai Chen, Xiaoting Wang, Yangong Chao, Dawei Liu

**Affiliations:** 1 Department of Critical Care Medicine, Peking Union Medical College Hospital, Chinese Academy of Medical Sciences and Peking Union Medical College, 1# Shuai Fu Yuan, Dong Cheng District, Beijing, 100730 China; 2 Department of Critical Care Medicine, Beijing Tongren Hospital, Capital Medical University, Beijing, 100730 China; 3 Department of Health Care, Peking Union Medical College Hospital, Chinese Academy of Medical Sciences and Peking Union Medical College, Beijing, 100730 China; 4 Pittsburgh Heart, Lung, Blood and Vascular Institute, University of Pittsburgh, School of Medicine, Pittsburgh, PA USA; 5 Department of Critical Care Medicine, The First Hospital of Tsing Hua University, Beijing, 100016 China

**Keywords:** Physician, Focused, Cardiac, Ultrasound, Critically ill

## Abstract

**Background:**

This study aimed to determine whether a focused 2-day cardiac ultrasound training course could enable physicians to obtain and interpret focused cardiac ultrasound (FCU) images from critically ill patients.

**Methods:**

We retrospectively reviewed the FCU images submitted by the physicians who attended a 2-day FCU training courses. Three experienced trainers reviewed the images separately. They determined whether the images were assessable and scored the images on an 8-point scale. They also decided whether the physicians provided correct responses for visual estimations of the left ventricular ejection fraction (LVEF) and right ventricle (RV) dilatation and septal motion.

**Results:**

Among the 327 physicians, 291 obtained images that were considered assessable (89%). The scores for parasternal short-axis view were lower than those obtained for other transthoracic echocardiographic views, *p* < 0.001. More physicians provided incorrect appraisals of LVEF than of RV dilatation and septal motion (19.9% vs. 3.1%, *p* < 0.001). The percentages of incorrect answers by LVEF category were as follows: 34.8% on images of LVEF < 30, 24.7% on images of LVEF 30–54, and 16.4% on images of LVEF ≥55%, *p* < 0.001. A logistic regression analysis showed that patients with abnormal LVEF were associated with physicians’ incorrect assessment of LVEF, with an odds ratio of 1.923 (95% confidence interval (CI):1.071–3.456, *p* = 0.029).

**Conclusions:**

A large proportion of physicians could obtain and interpret FCU images from critically ill patients after a 2-day training course. However, they still scored low on the parasternal short-axis view and were more likely to make an incorrect assessment of LVEF in patients with abnormal left ventricular systolic function.

## Background

It is increasingly recognized that cardiac ultrasound can play a pivotal role in the diagnosis and management of patients with shock or respiratory failure. Cardiac ultrasound examination allows the physicians to interpret the type of shock and differentiate the cause of hypoxia [1–3].

Probably the most common reason for requesting a cardiac ultrasound examination in the intensive care unit (ICU) is to assess left ventricular systolic function [4]. Prior researchers noted that the left ventricular ejection fraction (LVEF) estimated using eyeballing method was correlated significantly with those from formal quantitative methods [5]. Right ventricle (RV) function, which, if severely abnormal, can be assessed based on RV enlargement and paradoxical septal motion, is common in critically ill patients and has been attracting increasing attention [6–8].

Previous studies on critical ultrasound training noted that after a short training course, trainees demonstrated a significant improvement in their ability to obtain and interpret images [9–11]. However, most of the studies on focused cardiac ultrasound (FCU) training were based on human models, simulators and pathologic images prepared in advance. Few studies have reported the performance of physicians in terms of image acquisition and interpretation in critically ill patients after training. Thus, we investigated whether critical care physicians could obtain optimal FCU images and visually estimate LVEF and RV dilatation and septal motion in critically ill patients after a 2-day FCU training course.

## Methods

### Study design and participants

We enrolled critical care physicians who participated in a 2-day FCU training program sponsored by the Chinese Critical Ultrasound Study Group from May 2017 to May 2019. FCU images of critically ill patients uploaded by the physicians were retrospectively reviewed. Participants were excluded if they had previous experience with FCU or if they failed to upload the necessary images or image interpretations as requested by the training program.

The study was approved by the ethics committee of our institution. Informed consent was waived due to the retrospective and observational nature of this study.

### FCU training and the post-training assignment

The training program, which involved three hands-on practice sessions on adult human models and one session of image interpretation, aimed to enable the physicians to acquire basic views via transthoracic cardiac ultrasound and to obtain the basic ability to interpret the images. Fifty images, portraying various levels of LVEF and RV enlargement and paradoxical septal motion, were reviewed in an interactive manner during the image interpretation session. The details of the training course were described in a previous study [12].

To encourage the physicians to keep practicing and ensure they obtained the ability to acquire and interpret FCU images, every participant was asked to submit an assignment after the training; the assignment was to examine a critically ill patient during clinical practice and upload the FCU images to our website, together with the relevant information for the examined patient and the interpretation of the images. The physicians categorized the patients into three LVEF categories through the eyeballing method—poor (LVEF below 30%), moderate (LVEF 30–54%), or normal (LVEF ≥55%)—in accordance with cardiac ultrasound guidelines [13]. The physicians were also asked to assess the whether there was RV enlargement and paradoxical septal motion. The assignment was uploaded within 2 months after the training course and was deemed a necessary part of accreditation. All included patients provided authorization for their medical records to be reviewed for research. No personal information was included on the image or in the medical documents.

### Data collection

Three experienced trainers reviewed the uploaded images and interpretations. First, they determined if the images uploaded by the physicians, as a whole, fulfilled the criteria for making a heart function appraisal. Assessable images met the minimal criteria for diagnosis and displayed recognizable structures, in conformity with a semiquantitative method promulgated by the Emergency Ultrasound Standard Reporting Guidelines [14]. The physicians should obtain at least acceptable images of the parasternal long-axis view (PLAX), parasternal short-axis view (PSAX) and apical 4-chamber view (A-4CH). Then, the reviewers scored each FCU plane, including the PLAX, the PSAX, the A-4CH, the subcostal 4-chamber view (Subcostal 4CH), and the subcostal inferior vena cava view (Subcostal IVC), on an 8-point scale, according to the recommendations of prior FCU examinations [15, 16]. The specific scoring method for each plane is listed in Supplemental Table 1–5. The last step was to decide if the image interpretations of the physicians, including the LVEF estimation and whether there was RV enlargement and paradoxical septal motion, were correct. The reviewers performed the initial review work separately and then discussed the submissions about which there was disagreement. For borderline images, i.e., those showing LVEF close to 30% or 55%, if the reviewers’ assessment were still different after the discussion, answers in either adjacent range were viewed as correct. The reviewers were blinded to the physicians’ names.

### Statistical analysis

Statistical analysis was performed with the SPSS 13.0 statistical software package (SPSS Inc., Chicago, Illinois, USA). Continuous data are expressed as the mean ± SD or the median and the interquartile range. Categorical variables are presented as numbers and percentages. The normal distribution of the continuous values was assessed by the Kolmogorov-Smirnov test. Continuous variables were compared using the Kruskal-Wallis test. Categorical variables were compared with the chi-squared test or Fisher’s exact test, as appropriate. We performed a binary logistic analysis incorporating the physicians’ age, sex, professional rank, work experience in the ICU, and image obtainment score, and whether the examined patient was on mechanical ventilation (MV) support and whether the examined patient had abnormal LVEF to assess the independent factors for incorrect interpretation of LV dysfunction. The variables that had *p* < 0.25 in the univariable model were included in the multivariable analysis. All *p* values were two-tailed and statistical significance was defined as *p* < 0.05.

## Results

Three hundred twenty-seven critical care physicians participating in the FCU training program were included. The general characteristics of the physicians are displayed in Table 1.

**Table 1:** General characteristics of the physicians

Categories	Findings (*n* = 327)
Age (yr)	33 ± 5
Sex (male, %)	156 (47.7%)
Region^a^ (n, %)	
East China	89 (27.2%)
South China	45 (13.8%)
Central China	22 (6.7%)
North China	36 (11.0%)
Northwest China	11 (3.4%)
Southwest China	95 (29.1%)
Northeast China	29 (8.9%)
Professional ranks (n, %)	
Attending	46 (14.1%)
Fellow	140 (42.8%)
Residents	141 (43.1%)
ICU working experience (yr)	5 (3, 9)

^a^Region: where the trainees came from. ICU: intensive care unit

### Characteristics of the critically ill patients selected by the physicians

The mean age of the patients was 60 years old and 59.3% were men. The reasons for admission were respiratory failure (46.2%), shock (25.7%), respiratory failure plus shock (5.2%), cerebral diseases (11.0%), renal failure (3.1%) and other reasons (8.6%), e.g., high-risk surgery, multiple trauma, and metabolic disturbances. Patients on MV support accounted for 59.3% (Table 2).

**Table 2:** Characteristics of the patients

Categories	Findings (*n* = 327)
Age (yr)	60 ± 17
Sex (male, %)	194 (59.3%)
Reasons for ICU admission (n, %)	
Respiratory failure	151 (46.2%)
Shock	84 (25.7%)
Shock + Respiratory failure	17 (5.2%)
Cerebral diseases	36 (11.0%)
Renal failure	10 (3.1%)
Others	28 (8.6%)
MV support	194 (59.3%)

*ICU* intensive care unit, *MV* mechanical ventilation

### Image quality assessment

**Fig. 1:**
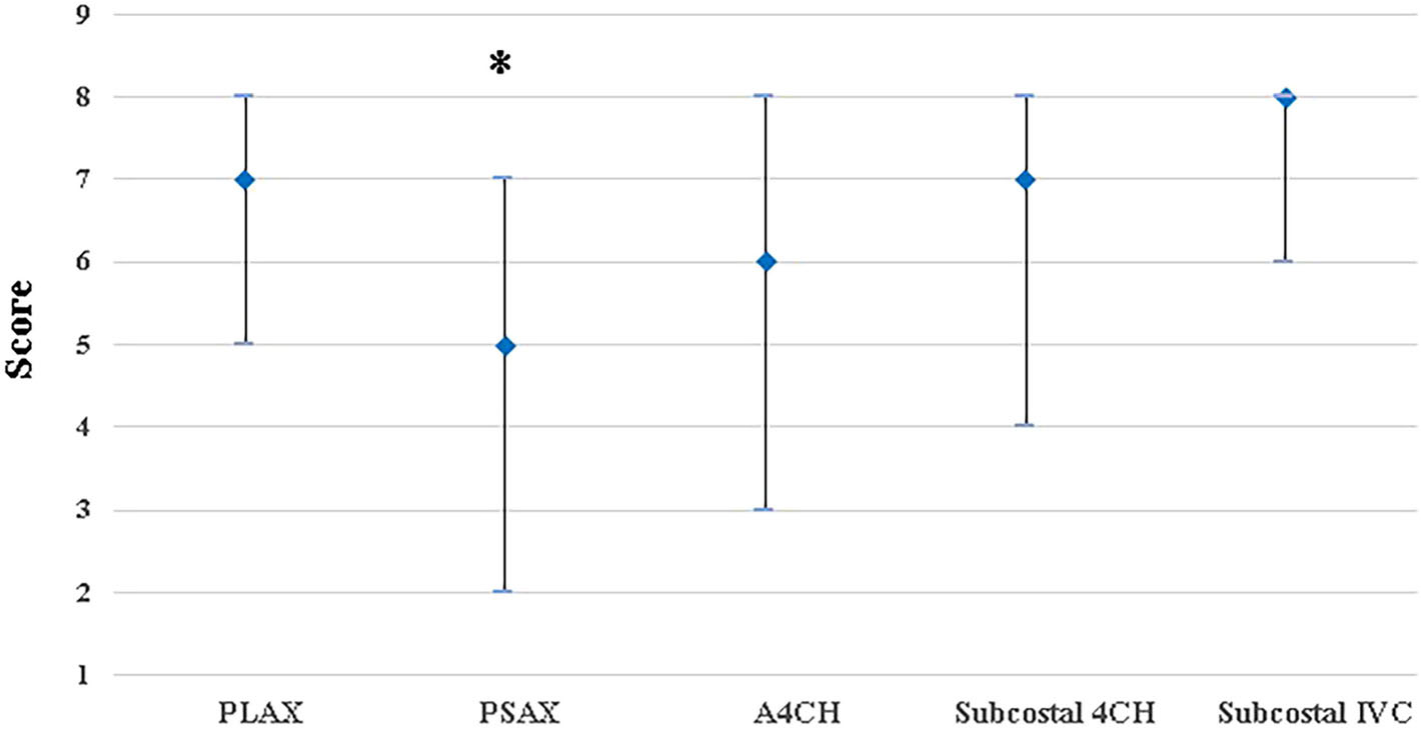
Image obtainment scores of the physicians from each FCU plane. The physicians achieved the lowest score from PSAX and achieved the highest score from Subcostal IVC, *p* < 0.001. FCU: focused cardiac ultrasound; PLAX: parasternal long-axis view; PSAX: parasternal short-axis view; A-4CH: apical 4-chamber view; Subcostal 4-CH: subcostal 4-chamber view; Subcostal IVC: subcostal inferior vena cava view.

Among the 327 physicians, 291 uploaded images that were considered assessable, i.e., the uploaded images that did not have discernable structures on the PLAX, the PSAX and the A4CH planes accounted for 11%. Regarding the scores obtained for each plane, physicians scored lowest score on the PSAX and highest on the Subcostal IVC, *p* < 0.001 (Table 3, Fig. 1).

**Table 3:** Image quality of the patients performed by the physicians

Categories	Findings (*n* = 327)
Assessable image	
Yes	291 (89.0%)
No	36 (11.0%)
Score of each plane	
PLAX	7 (5, 8)
PSAX	5 (2, 7)
A-4CH	6 (3, 8)
Subcostal 4CH	7 (4, 8)
Subcostal IVC	8 (6, 8)

*PLAX* parasternal long-axis view, *PSAX* parasternal short-axis view, *A-4CH* apical 4-chamber view, *Subcostal 4CH* subcostal 4-chamber view, *Subcostal IVC* subcostal inferior vena cava view

### Image interpretation assessment

The three reviewers were in agreement regarding the assessment of RV dilatation and septal motion. They were also consistent on 279/291 (95.8%) of the images related to LVEF. They had different opinions on 12 images; among them, 2 images that were close to 30% and had been categorized as LVEF 30–54% by the physicians were considered correct, and 10 images close to 55% and had been categorized as either 30–54% or ≥ 55% LVEF by the physicians were considered correct.

**Fig. 2:**
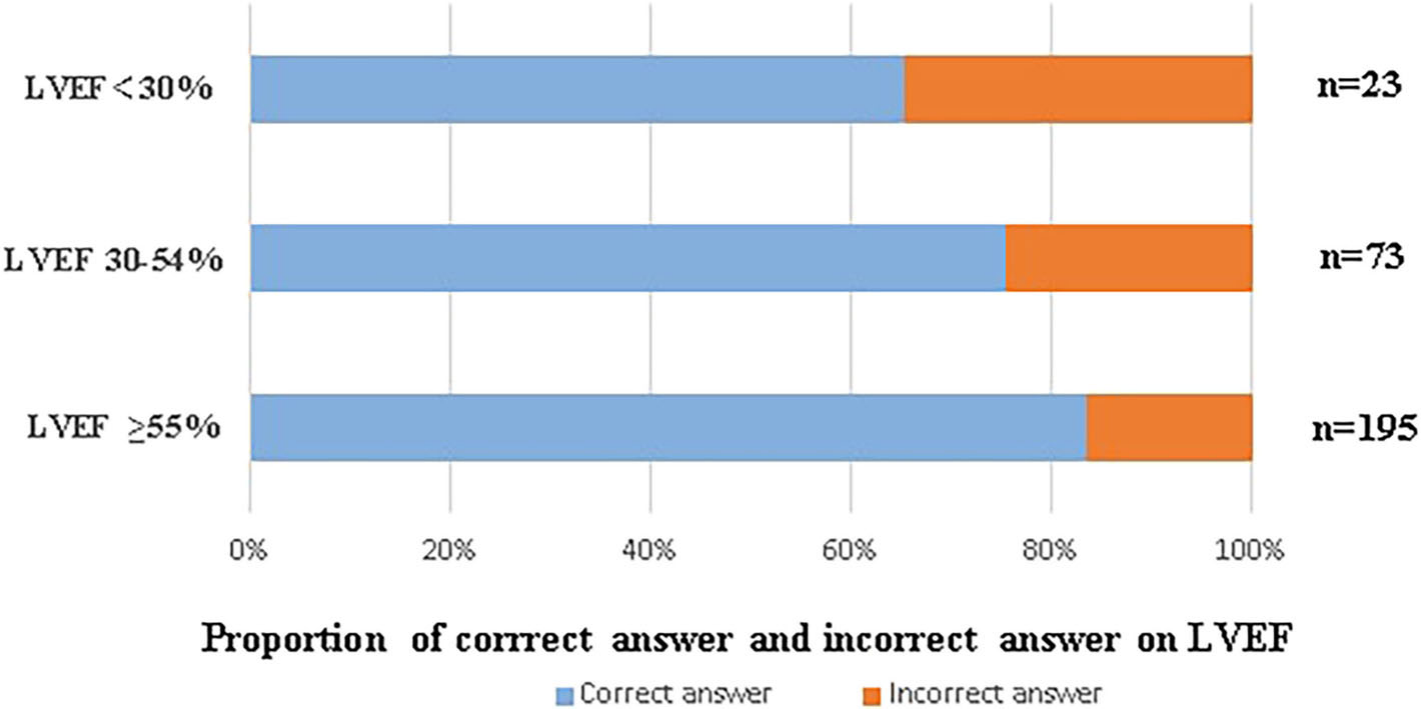
Proportion of correct and incorrect assessment of LVEF among patients in different LVEF categories. The proportion of incorrect answers in patients with LVEF< 30% was the highest, while the proportion of incorrect answers in patients with LVEF≥55% was the lowest, *p* < 0.001. LVEF: left ventricular ejection fraction.

A total of 58 physicians made an incorrect appraisal of the LVEF, and 9 physicians made an incorrect appraisal of RV function, *p* < 0.001. The percentages of physicians that gave incorrect answers, by category, are as follows: 34.8% on images of poor LVEF, 24.7% on images of moderate LVEF and 16.4% on images of normal LVEF, *p* < 0.001(Table 4, Fig. 2).

**Table 4:** Heart function assessment from the physicians

Categories	Incorrect answer
LVEF (*n* = 291)^a^	58 (19.9%)
< 30% (*n* = 23) ^b^	8 (34.8%)
30–54% (*n* = 73)	18 (24.7%)
≥ 55% (*n* = 195)	32 (16.4%)
RV enlargement and paradoxical septal motion (*n* = 291)	9 (3.1%)
Yes (*n* = 21)	4 (19.0%)
No (*n* = 270)	5 (1.9%)

^a^The proportion of wrong answers on LVEF was higher than on RV dysfunction, *p* < 0.001

^b^ The proportion of incorrect answers in patients with LVEF< 30% was the highest,

while the proportion of incorrect answers in patients with LVEF≥55% was the lowest, *p* < 0.001

*LVEF* left ventricular ejection fraction, *RV* right ventricle

In the logistic regression analysis, only patients with LV dysfunction were related to incorrect appraisals of LVEF, with an odds ratio of 1.923 (95% confidence interval (CI):1.071–3.456, *p* = 0.029) (Table 5).

**Table 5:** Risk factors for incorrect assessment on LV functions

Risk factor	OR	95%CI	*P*
Univariable analysis			
Physicians’ age	1.047	0.952–1.152	0.345
Physicians’ sex	0.997	0.564–1.761	0.990
Physicians’ professional rank	0.661	0.342–1.280	0.220
Physicians’ ICU work experience	0.995	0.929–1.066	0.895
Image obtainment score	0.993	0.949–1.040	0.768
Patient with MV support	0.773	0.425–1.405	0.397
Patient with LV dysfunction	1.999	1.106–3.613	0.022
Multivariable analysis			
Physicians’ professional rank	0.866	0.574–1.306	0.492
Patient with LV dysfunction	1.923	1.071–3.456	0.029

*ICU* intensive care unit, *MV* mechanical ventilation

## Discussion

This study found that after a 2-day FCU training course, a large proportion of critical care physicians were able to acquire basic cardiac ultrasound images from critically ill patients and interpret them with respect to visually estimating LVEF and RV function. Nonetheless, we also noticed that nearly one-third of trainees either provided suboptimal images or made incorrect assessments of basic heart function. They scored lowest scores on the PSAX and made more incorrect assessments of LVEF in patients with abnormal left ventricular systolic function.

Visual estimation of the LVEF is feasible and comparable to other quantitative methods and has several benefits, including time conservation and less dependence on image quality, both of which are crucial for critically ill patients [4, 5, 17]. The RV has complex structural geometry and quantitative RV assessment is more difficult, causing physicians to rely on visual estimates rather than quantitative assessments [18]. RV enlargement and paradoxical septal motion are the most commonly recommended parameters for detecting acute RV dysfunction [6, 19]. The trainees were able to make correct LVEF estimations in 81% of the patients and correctly assess RV function in 97% of the patients. Early detection of abnormal LVEF and RV dysfunction would clearly benefit the management of these patients. In this regard, optimal FCU images and the correct interpretation of them could enhance critical care physicians’ clinical skills, which is an advantage of this FCU training course.

The patients chosen by the trainees were all critically ill, reflecting the severity of patients in the physicians’ daily practice. The results showed that most of the patients were admitted for respiratory failure and/or shock. In addition to its utility in shock patients, cardiac ultrasound is also highly valuable for respiratory failure patients, who often have acute respiratory distress syndrome (ARDS), left ventricular dysfunction or pulmonary embolism, all of which greatly benefit from FCU in terms of diagnosis, differentiation and treatment monitoring [2, 20, 21]. Obtaining adequate FCU image quality in critically ill patients is often challenging due to MV, suboptimal patient positioning and indwelling catheters or drainage tubes [22]. We found that the proportion of assessable images reached 89% and that MV was not a risk factor for an incorrect assessment. Although we did not know how much time they would have or how many patients they would see, the physicians tried to finish the “homework”. We hypothesize that physicians’ free choice of patients contributed to the high proportion of assessable images.

Prior studies reported that emergency physicians or sonographers can master the skill of basic heart function and hemodynamic assessment during short-term cardiac ultrasound training [17, 23]. However, the individuals in those studies all had ultrasound experience. Melamed and his colleagues reported critical care physicians’ LVEF assessment ability. However, their study incorporated only 4 physicians, involved limited information about image acquisition and did not mention RV function appraisal [24]. In this study, we investigated the performance of FCU by critical care physicians in critically ill patients. The physicians were asked to provide images after the 2-day FCU training. Although this is a retrospective study, we report the performance of ICU physicians with no previous cardiac ultrasound experience on the FCU examination of critically ill patients.

Image obtainment lays the foundation for correct interpretation. Since the images and interpretations were uploaded as part of the test, we assume that they reflect the participants’ real abilities regarding FCU. The physicians showed relatively poor performance in terms of obtaining PSAX images, though this view is crucial in the assessment of regional wall motion abnormalities and paradoxical septal motion [18]. Some guidelines even recommend that the LVEF be measured from this view [25]. Therefore, more effort should be placed on image obtainment in the PSAX in future training programs.

We found that the physicians’ ability to perform heart function assessments was not related to the image obtainment score. Therefore, in addition to training the physicians to obtain images, more effort should also be placed on image interpretation. The main problem with the image interpretation was the visual assessment of the LVEF. In this study, the physicians were more likely to make incorrect LVEF estimations for patients with poor LVEF. Randazzo et al. observed that emergency physicians obtained higher scores on patients in the normal LVEF and poor LVEF categories after a three-hour training session in limited echocardiography [17]. However, their participants were all credentialed sonographers who could be expected be able to acquire this skill more rapidly than ultrasound beginners. Our previous study also found that ICU physicians obtained higher scores on images with poor LVEF during an image interpretation test, possibly because the different categories of LVEF, which were displayed on the same test, could be seen as a reference [12]. Hope et al. found that even untrained medical students could make acceptable LVEF estimations if they were provided an image reference [26]. We had expected better performance on poor LVEF images. The result reminded us that more emphasis should be placed on the poor LVEF category during future training programs. Another solution for beginners is to give them some standard images as references, which we speculate would improve their LVEF assessment ability. Muller et al. also pointed out that although visual estimation of LVEF was easy to learn, it also required practice, and the skill could be acquired more rapidly when a reference from other methods was available [27].

We found that the physicians’ age, sex, and work experience were not associated with their interpretation ability. This is in line with other researchers who noted that the visual estimation of ejection fraction can be learned by cardiac fellows and even medical students [26, 28]. Therefore, we conclude that clinical experience is not a prerequisite for the ability to be trained in evaluating basic heart function with FCU and that an FCU course could be taken in the early phase of medical education.

### Limitations

This study has several limitations. First, there was inevitable heterogeneity among the included critically ill patients. Both the patients’ intrinsic characteristics and the physicians’ own skills could have affected the physicians’ performance. However, since the images uploaded were an assignment for each physician, we assume they had the chance to choose patients who would allow them to show their real FCU examinations skills. Thus, we believe the physicians’ final performance was mainly determined by their own FCU skills rather than by the patients’ characteristics. As we mentioned above, the high proportion of assessable images in this study supported our assumption. Second, there was no “gold standard” for the image interpretation because we could not obtain the exact LVEF value of the examined patients. However, the reviewers were all highly experienced, and the final decisions were based on face-to-face discussions. The accuracy of estimating LVEF via eyeballing is quite high according to prior studies [5, 24]. Furthermore, we only needed to categorize the LVEF into three range categories. A previous study noted that experienced emergency physicians could assess LVEF with limited training [17]. Third, we were not able to differentiate patients with LV diastolic dysfunction or chronic RV dysfunction due to the nature of this basic FCU examination. Despite these limitations, we believe that the results of this study might shed light on the direction of future FCU training for critical care physicians.

## Conclusions

After a 2-day FCU training course, a large proportion of critical care physicians displayed acceptable abilities to obtain images and perform basic heart function assessment in critically ill patients. However, they still had low scores in the obtainment of the parasternal short-axis view and were more likely to make an incorrect assessment of the LVEF in patients with abnormal left ventricular systolic function.

## Supplementary information

**Additional file 1: Table S1**. Criteria of Parasternal long-axis view. **Table S2.** Criteria of parasternal short-axis view. **Table S3.** Criteria of Apical 4-chamber. **Table S4.** Criteria of subcostal 4-chamber view. **Table S5.** Criteria of subcostal inferior vena cava.

## Data Availability

Data is available from the corresponding author on reasonable request.

## Abbreviations

FCU: Focused cardiac ultrasound
LVEF: Left ventricular ejection fraction
RV: Right ventricle
LV: Left ventricle
PLAX: Parasternal long-axis view
PSAX: Parasternal short-axis view
A-4CH: Apical 4-chamber view
Subcostal 4CH: Subcostal 4-chamber view
Subcostal IVC: Subcostal inferior vena cava view
MV: Mechanical ventilation
