# Supplementary material for: Physicians’ abilities to obtain and interpret focused cardiac ultrasound images from critically ill patients after a 2-day training course
Source: BMC Cardiovasc Disord. 2020 Mar 30;20:151. doi: 10.1186/s12872-020-01423-2 (PMC7106613; doi:10.1186/s12872-020-01423-2)
Supplement: Supplementary file 1 — Additional file 1: Table S1. Criteria of Parasternal long-axis view. Table S2. Criteria of parasternal short-axis view. Table S3. Criteria of Apical 4-chamber. Table S4. Criteria of subcostal 4-chamber view. Table S5. Criteria of subcostal inferior vena cava. [file 12872_2020_1423_MOESM1_ESM.doc]

Supplemental table 1. Criteria of Parasternal long-axis view

| Content | Score |
| --- | --- |
| Adequate depth | 1 |
| Adequate gain | 1 |
| Interventricular septum and posterior wall parallel with each other | 2 |
| Aortic valve and ascending aorta being bisected | 2 |
| Absence of apex | 2 |
| Total | 8 |

Supplemental table 2. Criteria of parasternal short-axis view

| Content | Score |
| --- | --- |
| Adequate depth | 1 |
| Adequate gain | 1 |
| Left ventricle displayed in circular shape | 2 |
| Mitral valve, apex area view being shown as the probe being tiled | 2 |
| Presence of two papillary muscles | 2 |
| Total | 8 |

Supplemental table 3. Criteria of Apical 4-chamber

| Content | Score |
| --- | --- |
| Adequate depth | 1 |
| Adequate gain | 1 |
| Interventricular septum being vertical | 2 |
| Presence of four chamber | 2 |
| Absence of apex moving downward | 2 |
| Total | 8 |

Supplemental table 4. Criteria of subcostal 4-chamber view

| Content | Score |
| --- | --- |
| Adequate depth | 1 |
| Adequate gain | 1 |
| Presence of four chamber | 2 |
| Presence of mitral valve and tricuspid valve | 2 |
| Interatrial septum clearly shown | 2 |
| Total | 8 |

Supplemental table 5. Criteria of subcostal inferior vena cava

| Content | Score |
| --- | --- |
| Adequate depth | 1 |
| Adequate gain | 1 |
| Presence of IVC into right atrium | 2 |
| Presence of hepatic vein into IVC | 2 |
| Anterior and posterior wall of IVC paralleled | 2 |
| Total | 8 |

IVC: inferior vena cava
